# Supplementary material for: White Light-Photolysis for the Removal of Polycyclic Aromatic Hydrocarbons from Proximity Firefighting Protective Clothing
Source: Int J Environ Res Public Health. 2022 Aug 15;19(16):10054. doi: 10.3390/ijerph191610054 (PMC9407878; doi:10.3390/ijerph191610054)
Supplement: Supplementary file 1 [file ijerph-19-10054-s001.zip › ijerph-1782937-supplementary.pdf]

International Journal of
Environmental Research and Public Health

Paper - Supplementary material

White Light-Photolysis for the Removal of Polycyclic Aromatic Hydrocarbons from Proximity Firefighting Protective Clothing

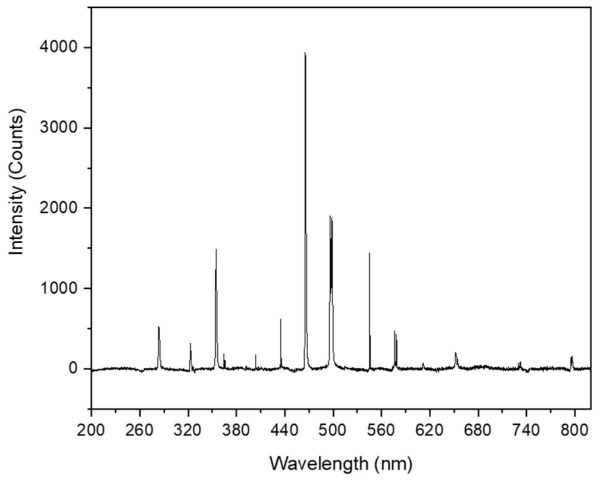

Figure S1. Emission spectrum of 250 W mercury vapor and tungsten filament white light.

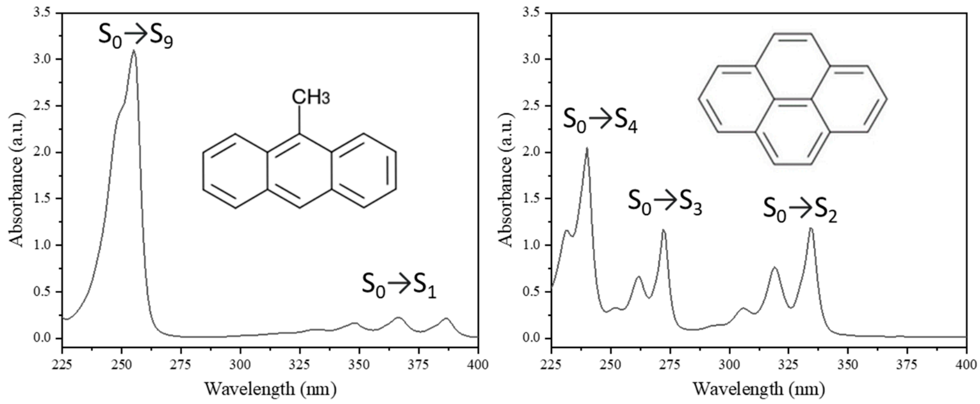

Figure S2. spectrum of 250 W mercury vapor and tungsten filament white light.

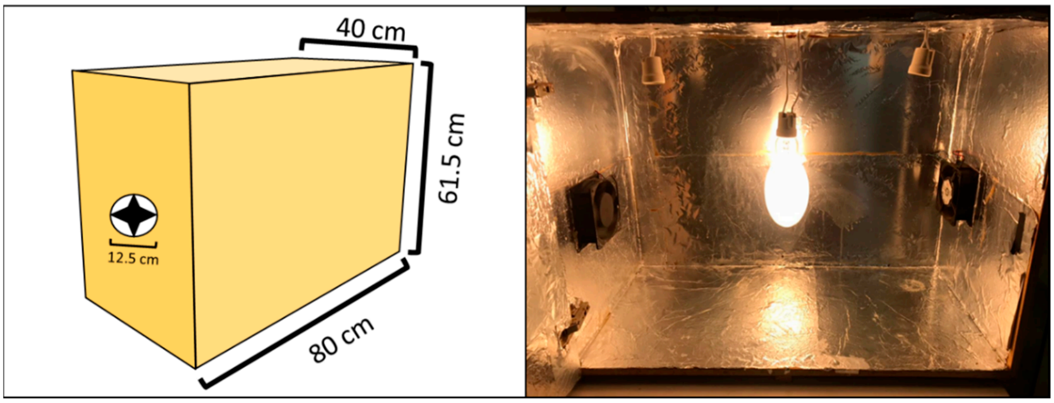

Figure S3. Photoreactor equipped white light.

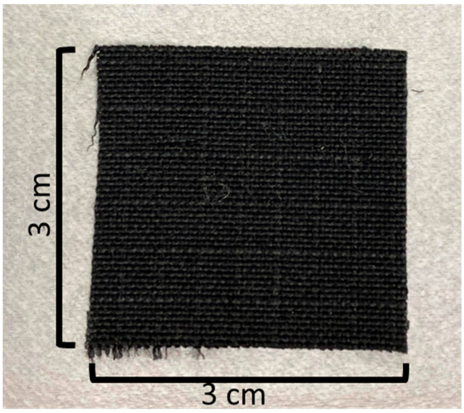

Figure S4. Samples of fabric from the outer shell of the firefighting uniform with composition of 58% para-aramid (kevlar®), 40% meta-aramid (nomex®) and 2% carbon, from Unishell®.

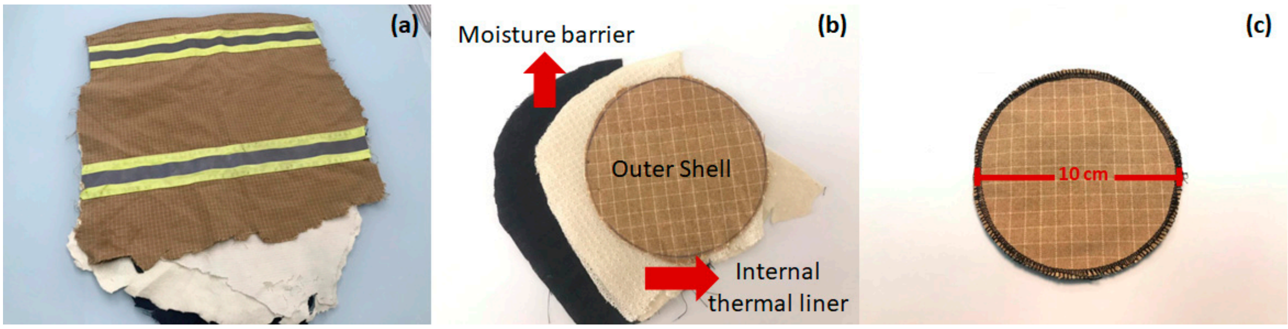

Figure S5. Samples of three-layer fabrics from Brazilian firefighters' protective clothing. **(a)** jacket pieces, **(b)** three-layer fabrics samples **(c)** disk with the three layers sewn together.

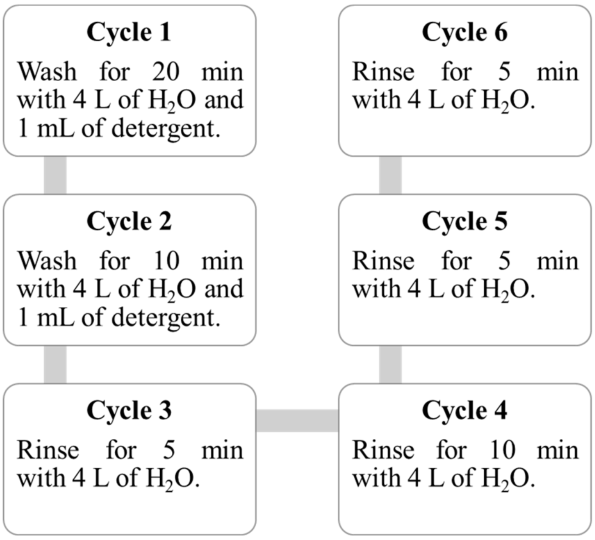

Figure S6. Scheme of three-layer fabric laundering decontamination.

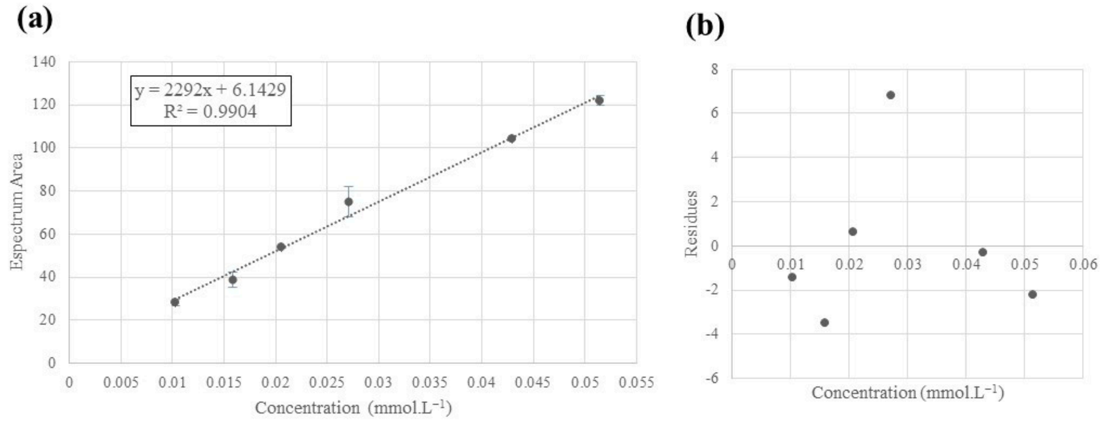

Figure S7. UV/VIS **(a)** calibration curve for PAHs solution and **(b)** residual graph.

Table S1. UV/VIS ANOVA table for PAHs solution.

Source	*Sum of Squares*	*Degrees of Freedom*	*Mean Squares*
Regression	6780.34	1	6780.34
Residual	65.96	4	16.49
Lack of fit	65.96	4	16.49
Total	6846.30	5	1369.268

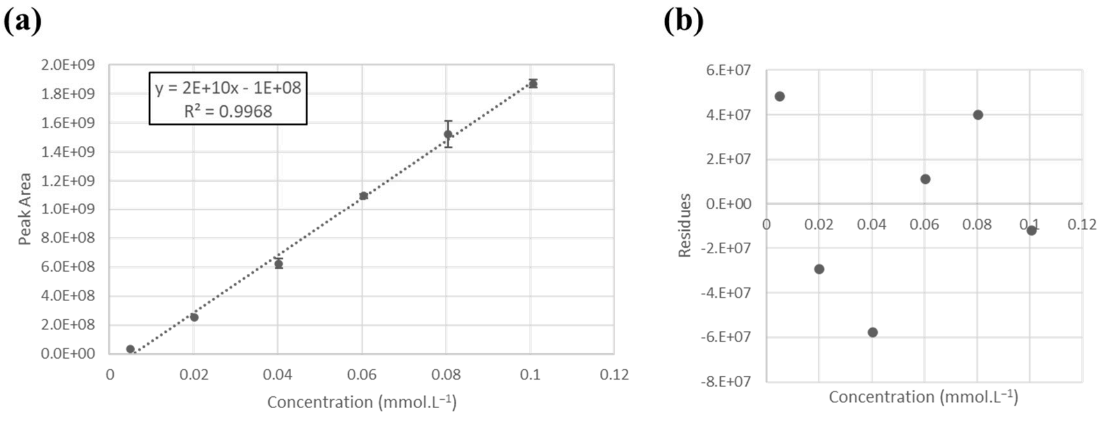

Figure S8. GC/MS **(a)** calibration curve for PAHs solution and **(b)** residual graph for 9-methylanthracene.

Table S2. GC/MS ANOVA table for 9-methylanthracene.

Source	*Sum of Squares*	*Degrees of Freedom*	*Mean Squares*
Regression	2.65E+18	1	2.65E+18

Residual	1.36E+16	4	3.39E+15
Lack of fit	1.36E+16	4	3.39E+15
Total	2.67E+18	5	5.33E+17

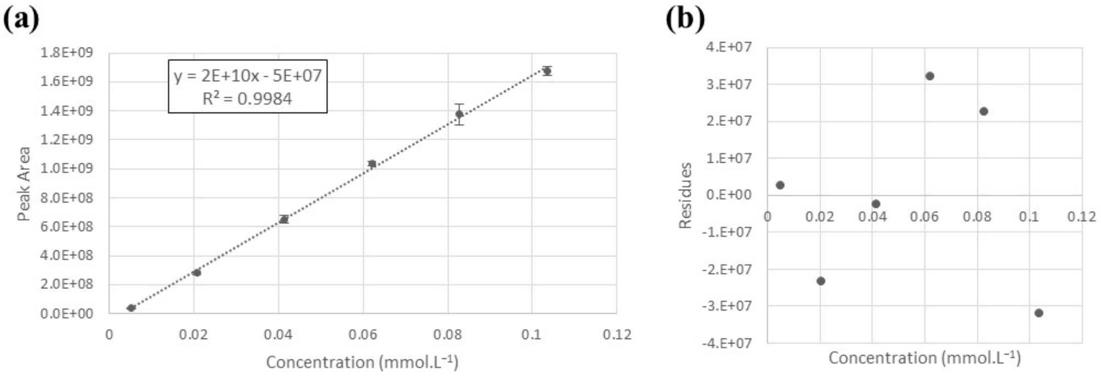

Figure S9. GC/MS **(a)** calibration curve for PAHs solution and **(b)** residual graph for pyrene.

Table S3. GC/MS ANOVA table for pyrene.

Source	*Sum of Squares*	*Degrees of Freedom*	*Mean Squares*
Regression	2.00E+18	1	2.00E+18
Residual	3.12E+15	4	7.81E+14
Lack of fit	3.12E+15	4	7.81E+14
Total	2.01E+18	5	4.02E+17

Table S4. Limits of detection (LD) and quantification (LQ), in mmol.L-1, obtained by the analytical curves for the analytes using GC/MS.

	**9-methylanthracene**	**Pyrene**
**LD**	0.00021 mmol.l^{-1}	0.00016 mmol.l^{-1}
**LQ**	0.00071 mmol.l^{-1}	0.00052 mmol.l^{-1}

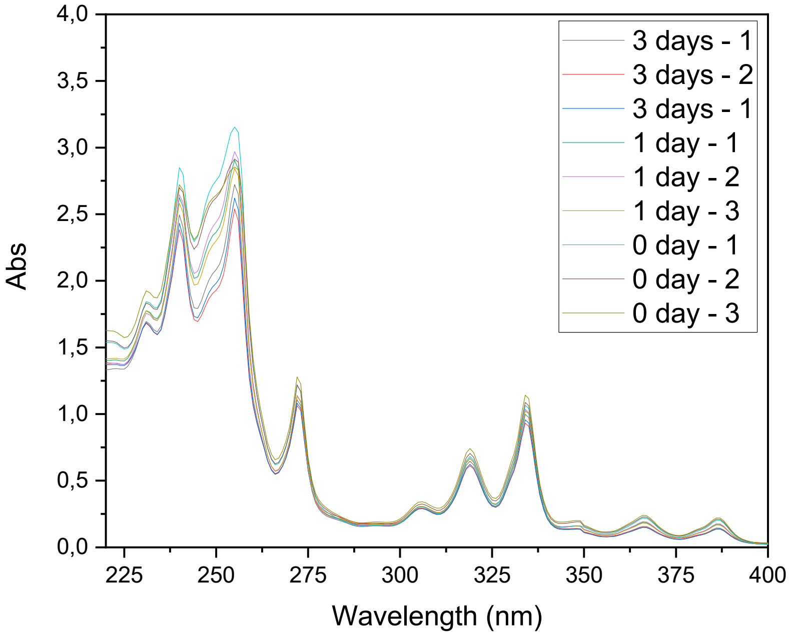

Figure S10. UV/VIS spectra of experiments performed in 0, 1 and 3 days with BLK 0% of H_2O_2.

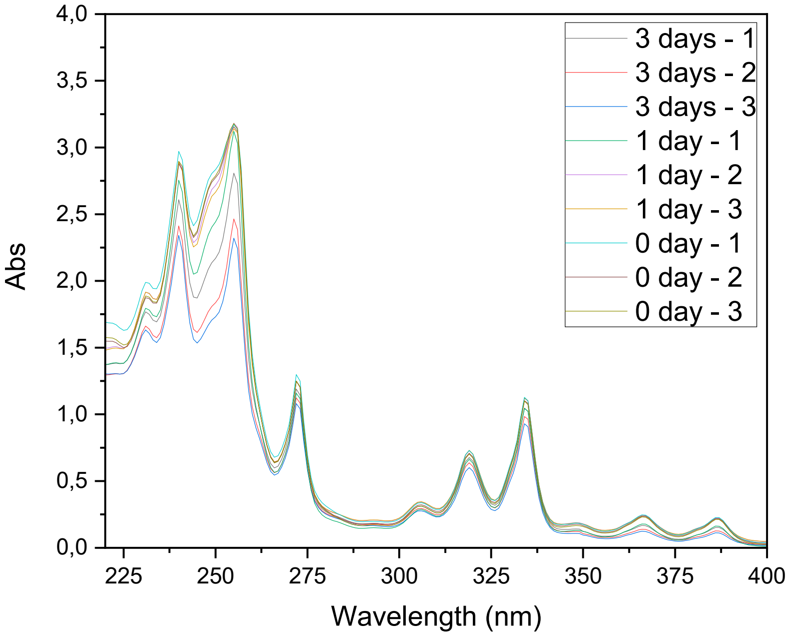

Figure S11. UV/VIS spectra of experiments performed in 0, 1 and 3 days with BLK 0.35% of H_2O_2.

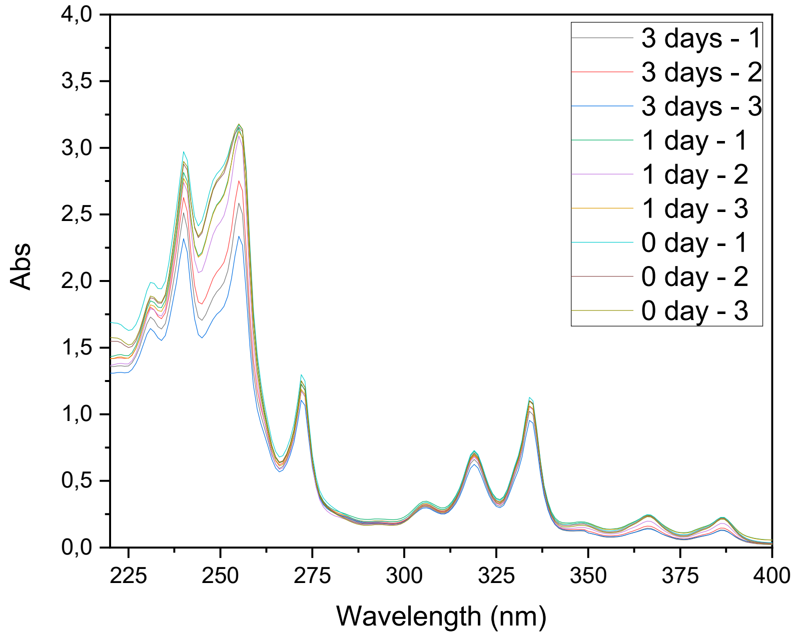

Figure S12. UV/VIS spectra of experiments performed in 0, 1 and 3 days with BLK 3.5% of H_2O_2.

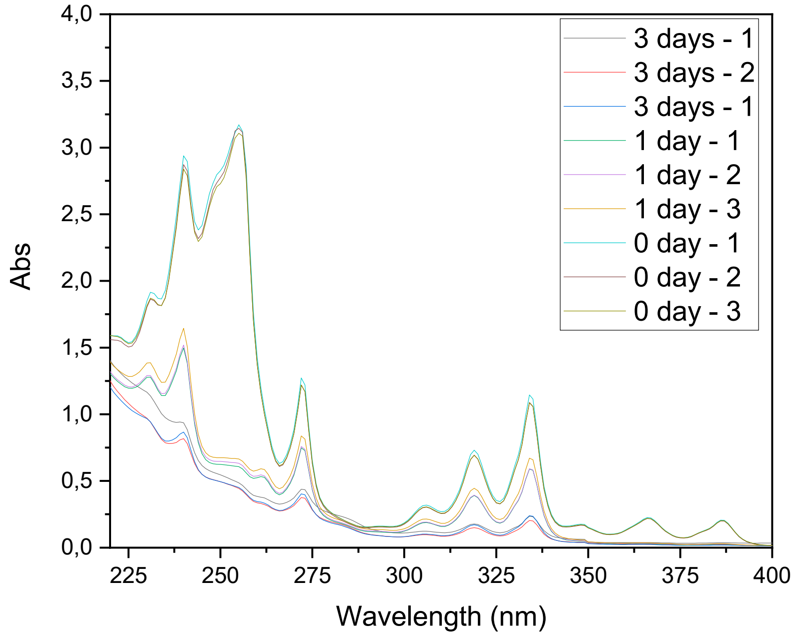

Figure S13. UV/VIS spectra of experiments performed in 0, 1 and 3 days with WLP 0% of H_2O_2.

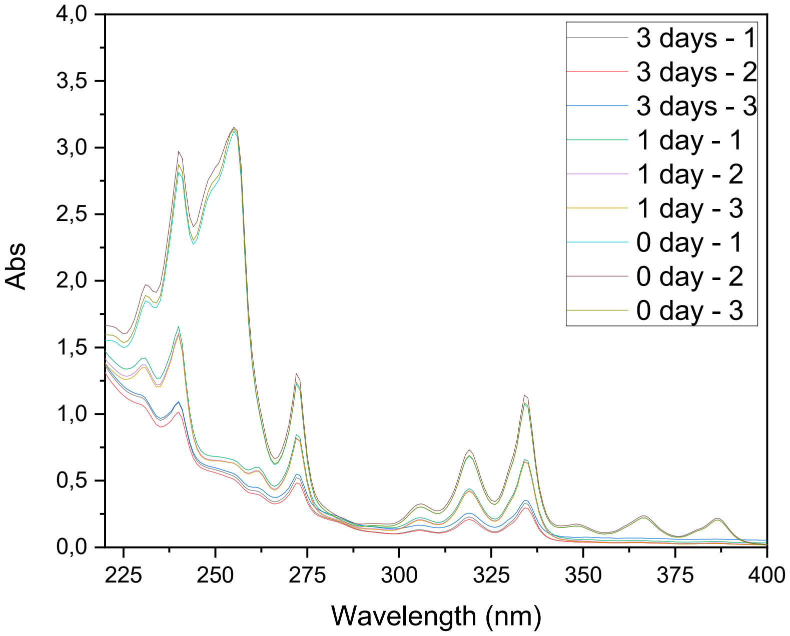

Figure S14. UV/VIS spectra of experiments performed in 0, 1 and 3 days with WLP 0.35% of H_2O_2.

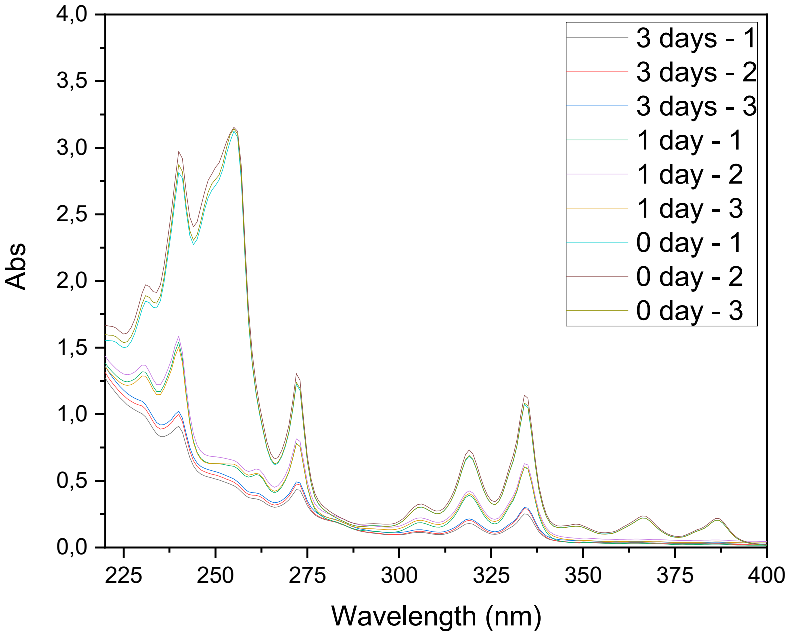

Figure S15. UV/VIS spectra of experiments performed in 0, 1 and 3 days with WLP 3.5% of H_2O_2.

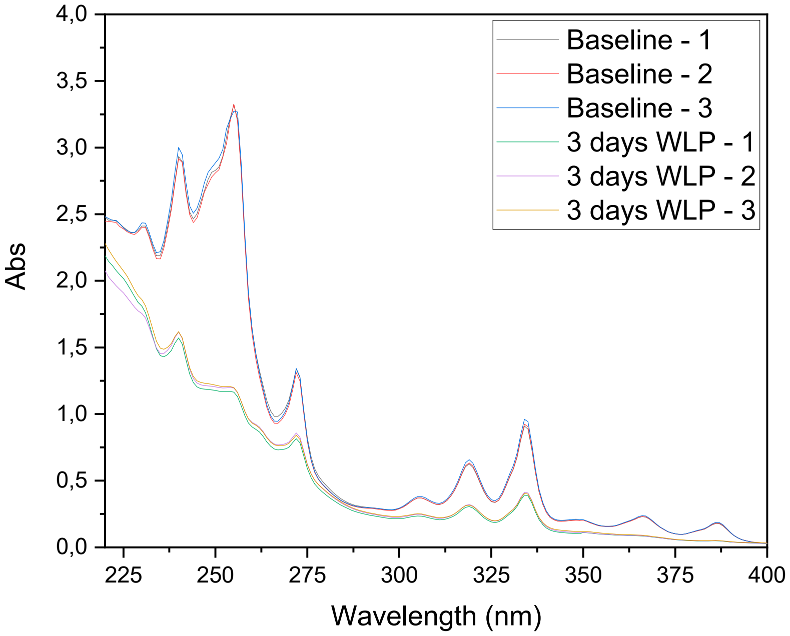

Figure S16. UV/VIS spectra of decontamination procedure performed for WLP decon for 3 days with 0% of H_2O_2.

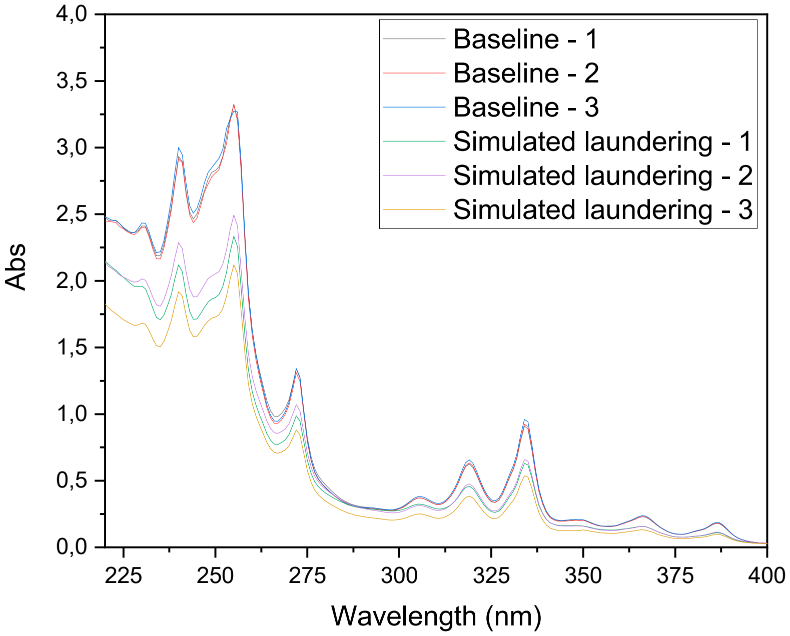

Figure S17. UV/VIS spectra of decontamination procedure performed for simulated laundering decon.

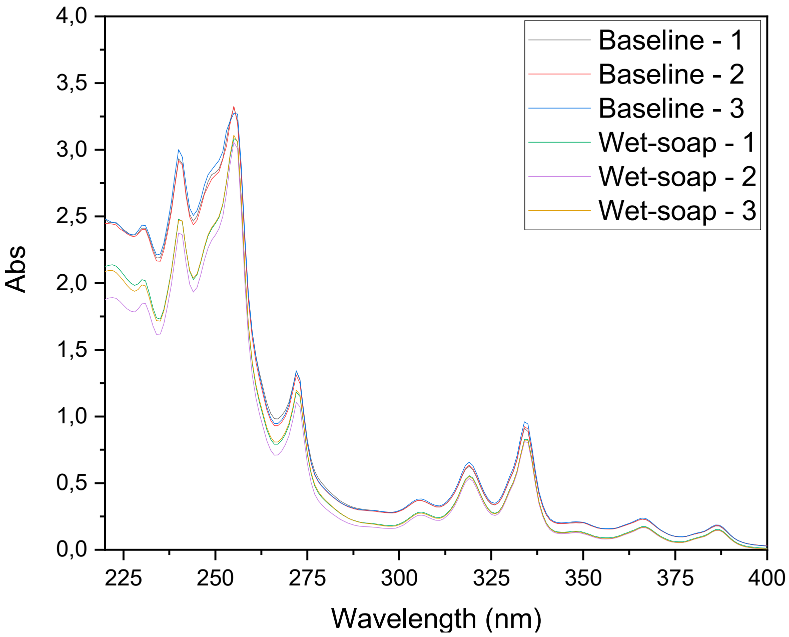

Figure S18. UV/VIS spectra of decontamination procedure performed for wet-soap brushing decon.

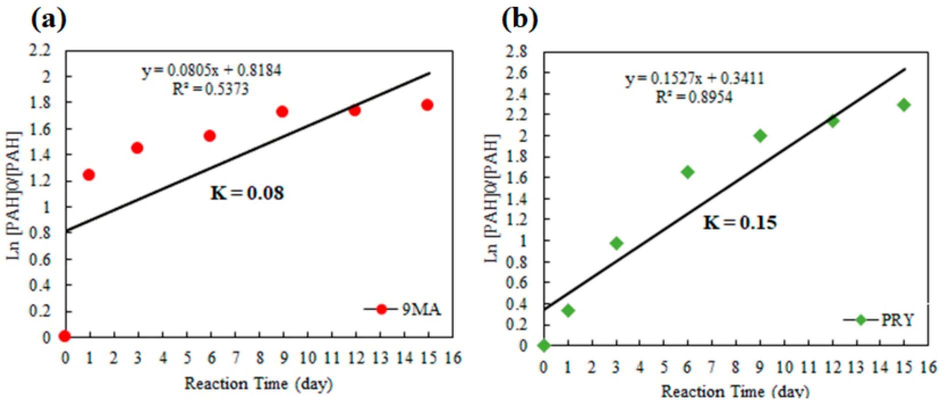

Figure S19. Pseudo first order rate constants for WLP 0% of H_2O_2 reaction of **a)** 9-methylanthracene and **b)** pyrene degradation.

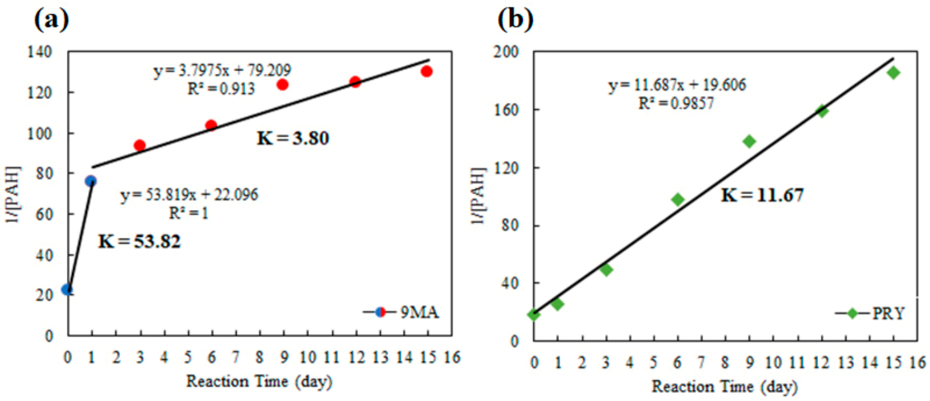

Figure S20. Pseudo second order rate constants for WLP 0% of H_2O_2 reaction of **a)** 9-methylanthracene and **b)** pyrene degradation.

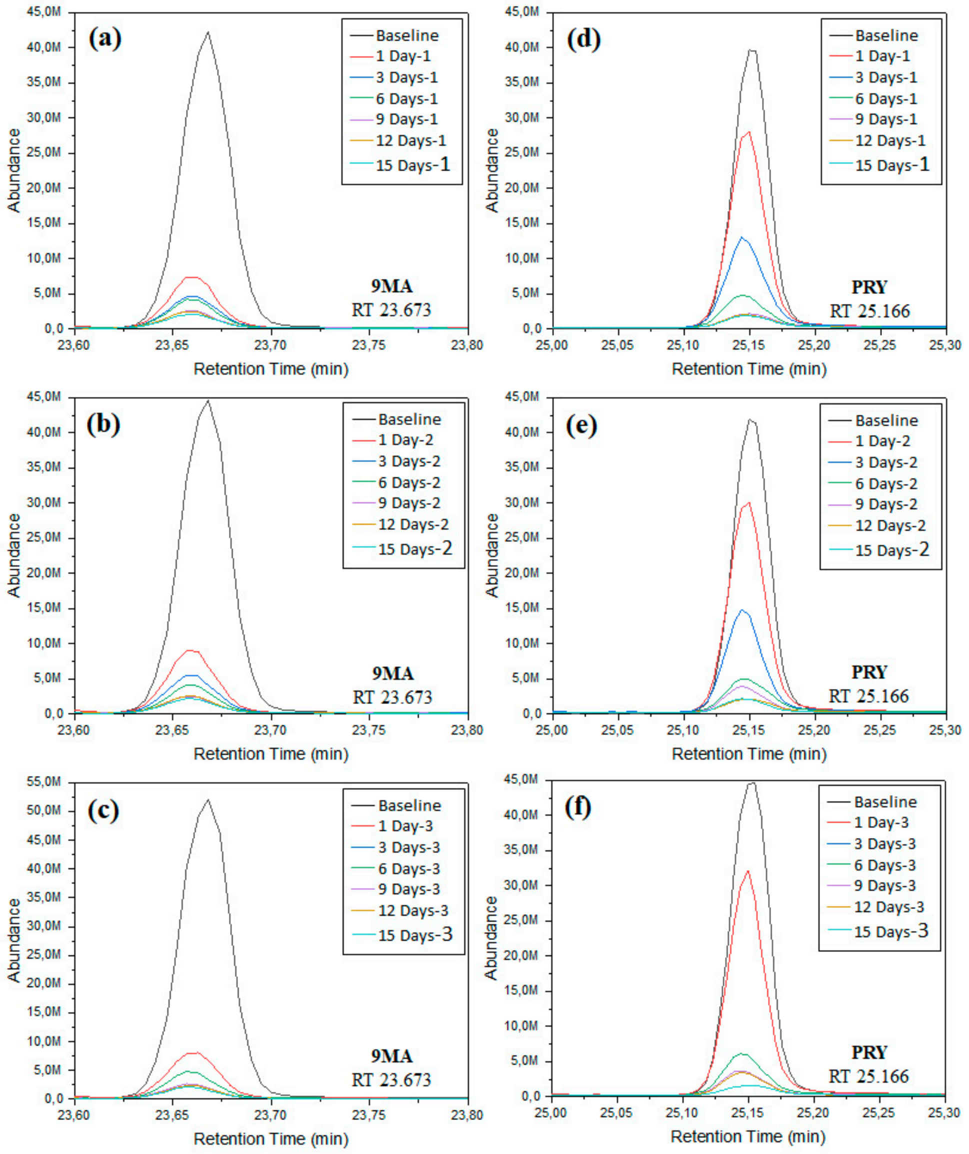

Figure S21. Chromatograms replicas of WLP 0% H_2O_2 experiments for the analyte 9-methylanthracene **(a-c)** obtained in the retention time of 23.673 min and for the analyte pyrene **(d-f)** obtained in the retention time of 25.166 min.

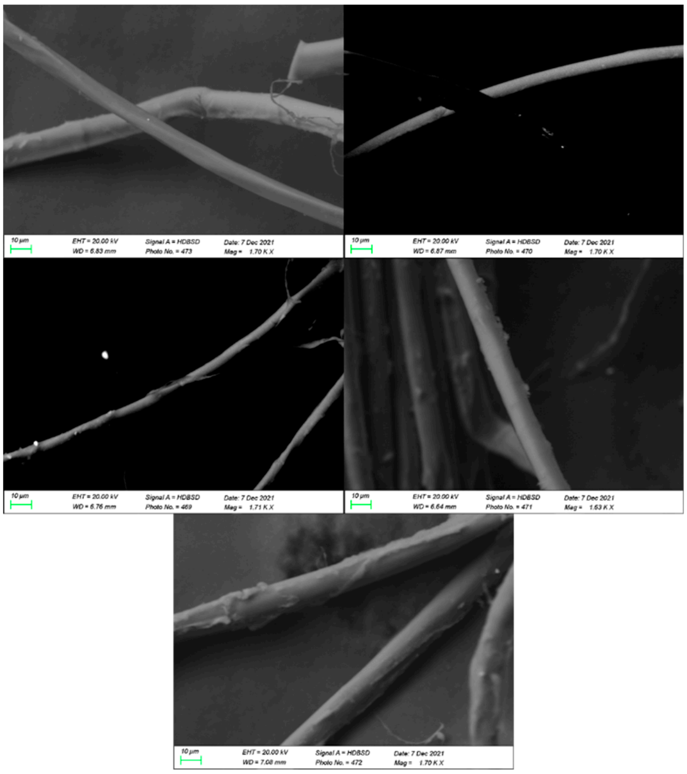

Figure S22. Micrographs of fibers before treatment.

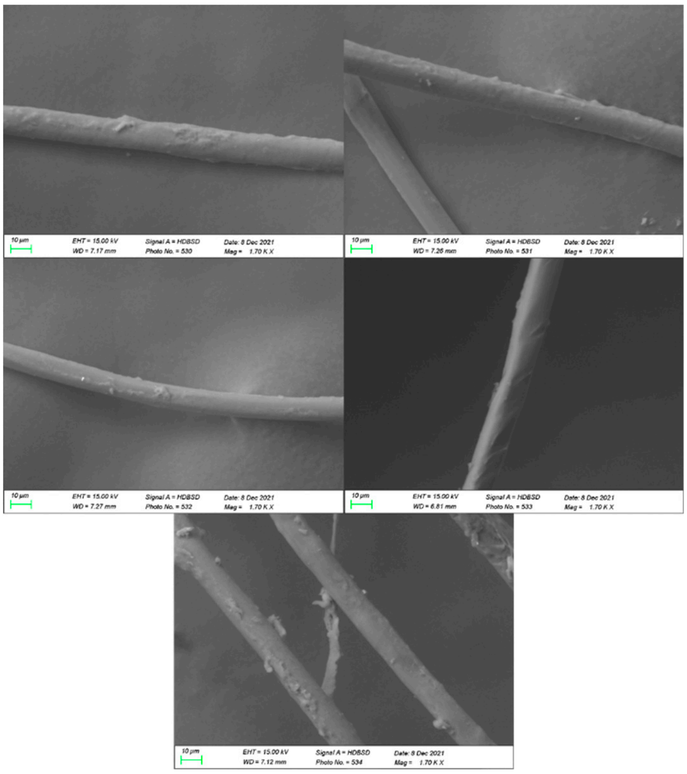

Figure S23. Micrographs of fibers after treatment with BLK 0% of H_2O_2.

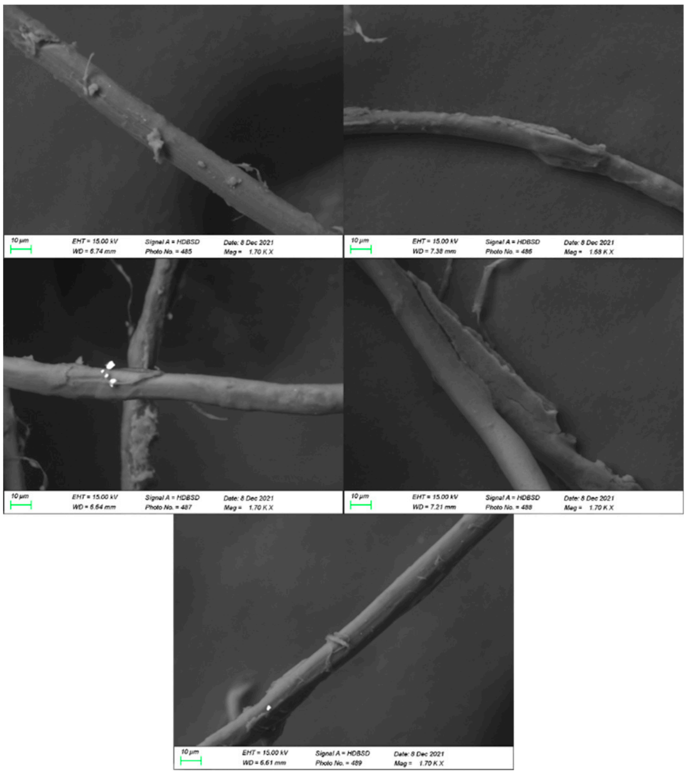

Figure S24. Micrographs of fibers after treatment with BLK 0.35% of H_2O_2.

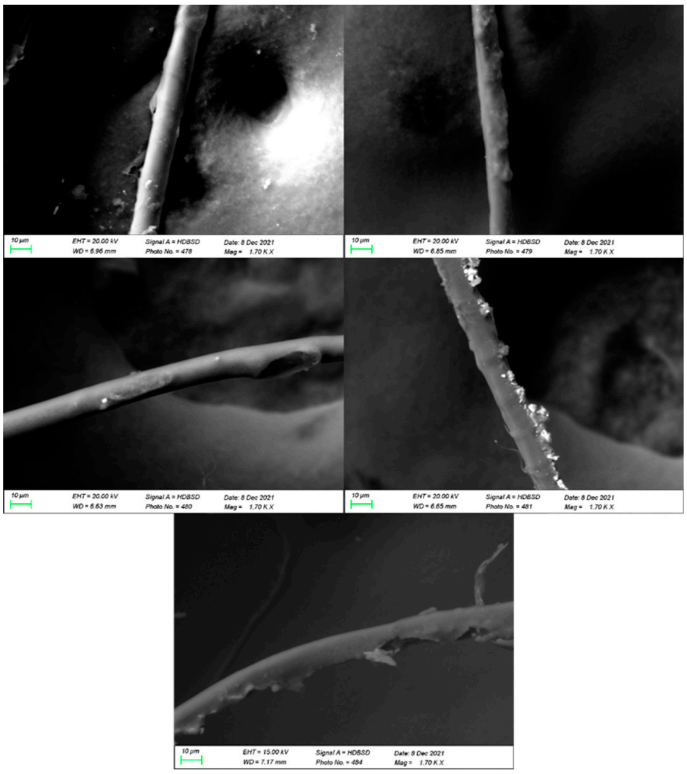

Figure S25. Micrographs of fibers after treatment with BLK 3.5% of H_2O_2.

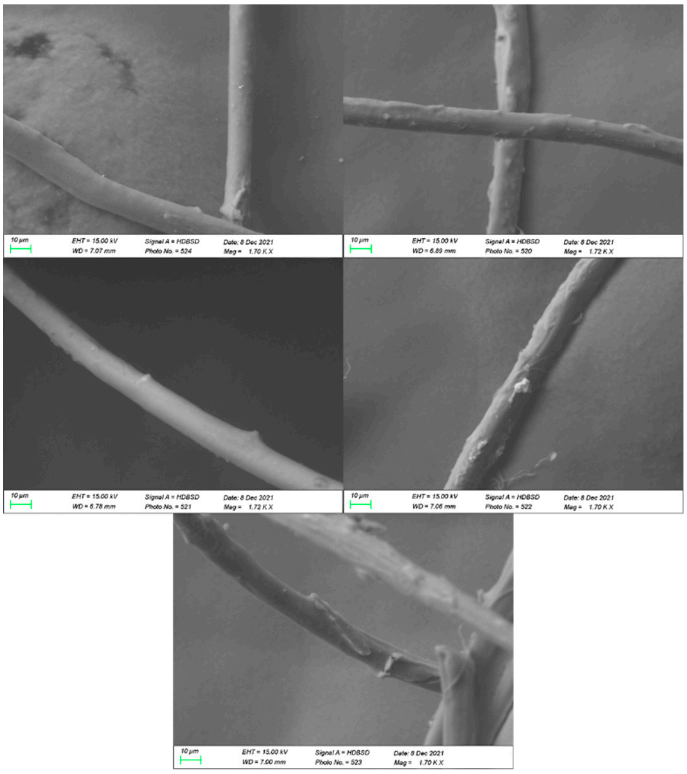

Figure S26. Micrographs of fibers after treatment with WLP 0% of H_2O_2.

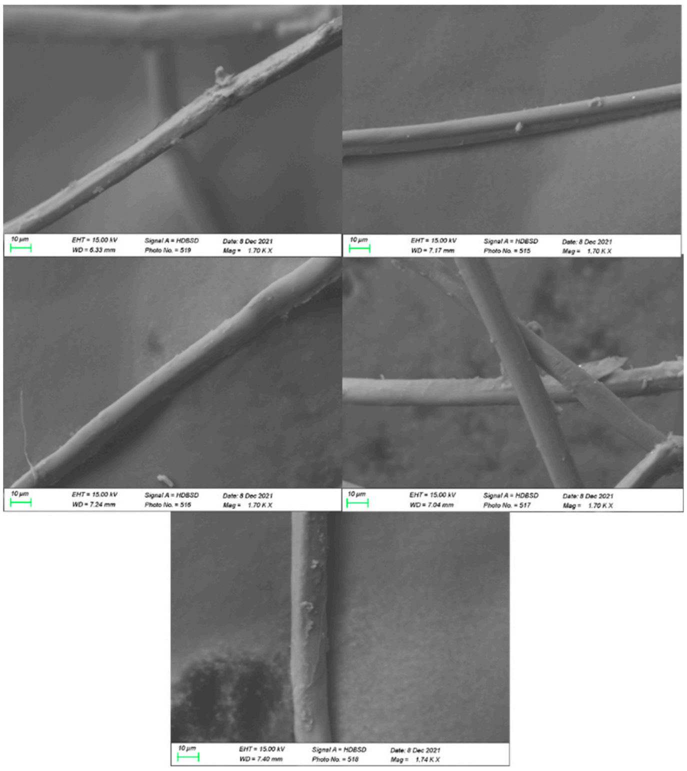

Figure S27. Micrographs of fibers after treatment with WLP 0.35% of H_2O_2.

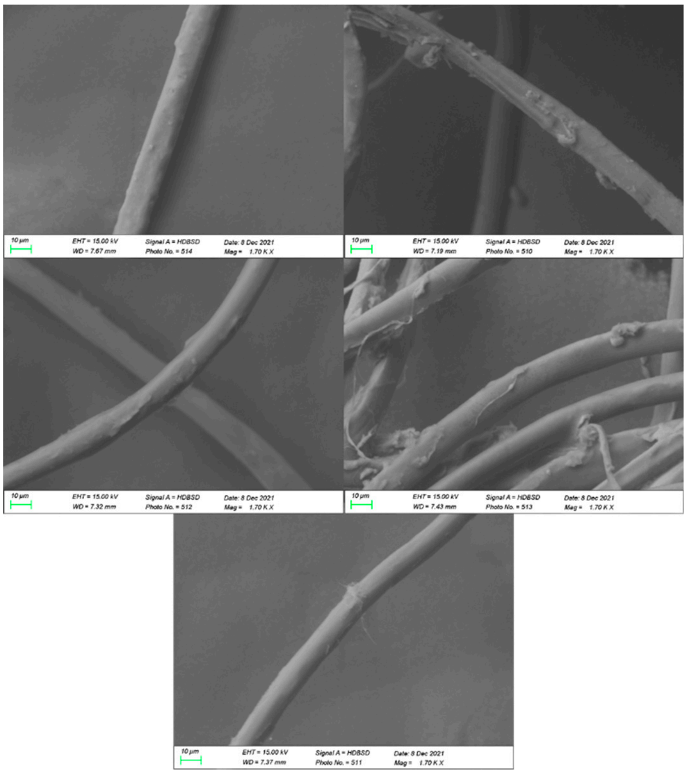

Figure S28. Micrographs of fibers after treatment with WLP 3.5% of H_2O_2.

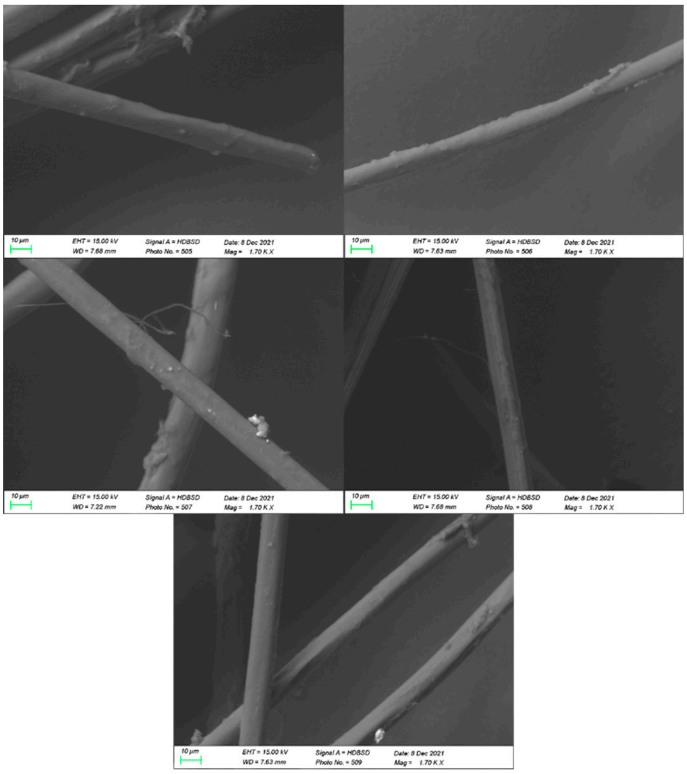

Figure S29. Micrographs of fibers after treatment with WLP 0% of H_2O_2 for 30 days.

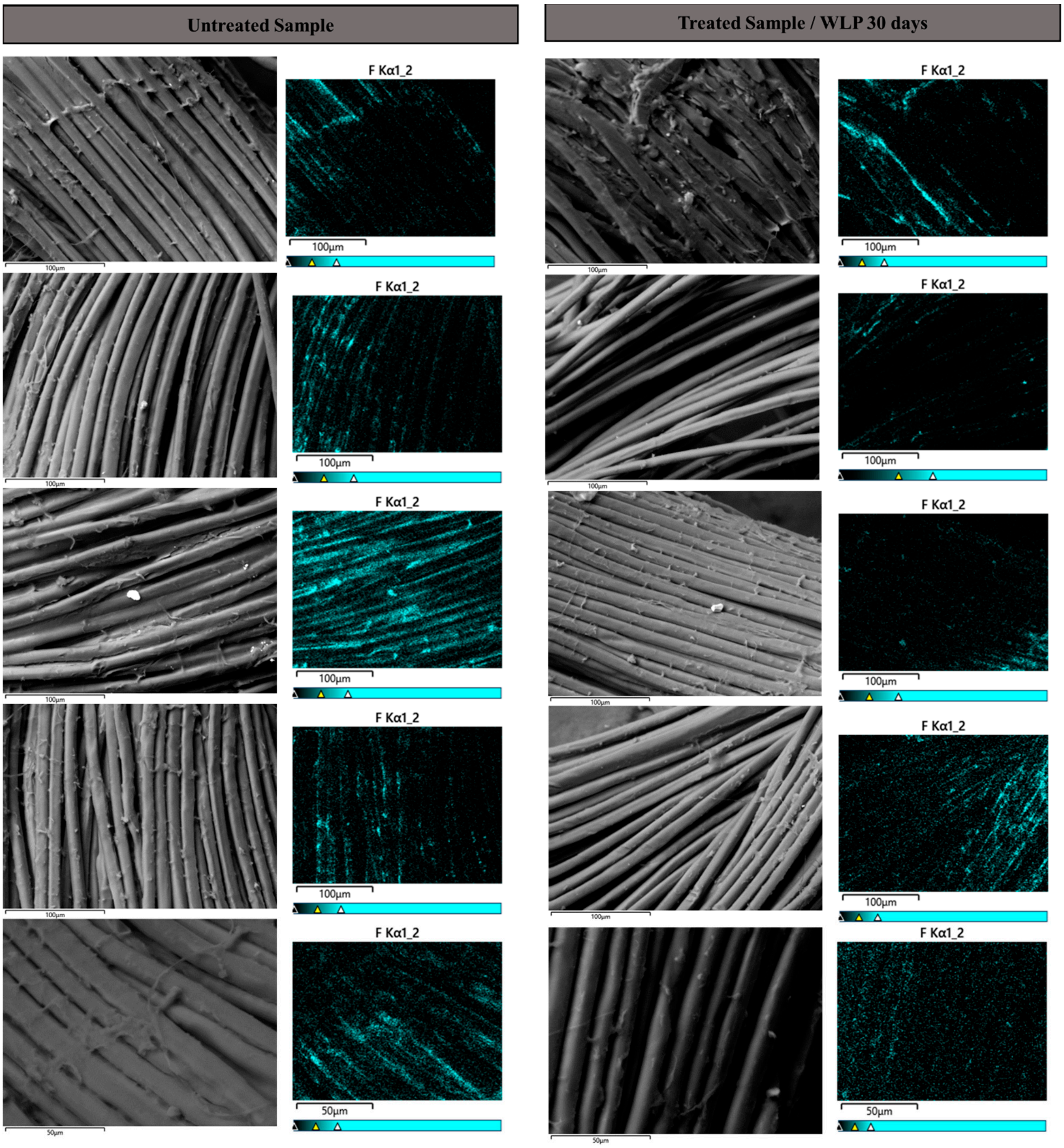

Figure S30. Compositional maps for fluorine for the untreated sample and for the treated sample with WLP and 0% H_2O_2 for 30 days.
